# RegulatorDB: a resource for the analysis of yeast transcriptional regulation

**DOI:** 10.1093/database/bax058

**Published:** 2017-08-03

**Authors:** John A. Choi, John J. Wyrick

**Affiliations:** 1 School of Molecular Biosciences; 2 Center for Reproductive Biology, Washington State University, Pullman, WA 99164, USA

## Abstract

Mutant expression profiles have been published for nearly all the nonessential regulators in yeast, yet there is a need for improved analysis and visualization tools to analyze these data and integrate it with complementary protein-DNA binding data. The RegulatorDB database contains mutant expression profiles and DNA binding data for more than 900 and 250 yeast regulators, respectively. RegulatorDB provides web-based tools to visualize the effects of each mutant regulator on the expression of individual genes or user-selected gene sets, and identify regulators whose targets are enriched in user-selected gene sets. The database can be queried to search for targets of single or multiple regulators. Regulatory networks can be constructed and visualized that include multiple classes of regulators and multiple regulatory layers, including regulator DNA binding data. In summary, RegulatorDB is a powerful resource for the study of yeast gene regulation, from the level of individual genes up to genome-scale networks.

**Database URL:** http://wyrickbioinfo2.smb.wsu.edu/RegulatorDB

## Introduction

Gene transcription in eukaryotic cells is controlled by multiple categories of regulator proteins, including sequence-specific DNA binding proteins, co-activators, chromatin factors, and kinase and phosphatase enzymes. Often multiple regulators from each of these categories will cooperate to control the expression of a single gene.

Mutant expression profiles of candidate regulators are an important resource for studies of yeast gene regulation. Recently, a large set of mutant expression profiles (for >700 distinct yeast gene deletion mutants) have been published for nearly all of the known, nonessential regulators in yeast (1–3). Importantly, these studies have been performed using self-consistent and uniform growth conditions, experiment procedures, and data analysis methods. However, much of these data (i.e. the chromatin regulator, kinase and phosphatase and ′other′ regulator data sets) are not currently available in yeast gene regulation databases (4, 5). Chromatin immunoprecipitation-microarray (ChIP-chip) experiments have also been extensively used to identify DNA bound target genes for many transcription factors [e.g. (6, 7)], but these data could be better integrated with mutant expression profiles. We have developed the RegulatorDB database to integrate these data in a single online portal and provide tools to analyze the mutant expression profiles and DNA-binding targets for nearly all regulators in *Saccharomycescerevisiae*.

## Materials and methods

RegulatorDB contains mutant expression profiles for 165 chromatin regulators, 142 kinases and phosphatases, 68 transcription factors and 418 other regulators. Mutant expression profiles characterized under different growth conditions (yeast extract peptone dextrose (YPD) instead of synthetic complete (SC) media) are included for 258 transcription factors and co-activators (8, 9). These data were compiled from published sources (1–3, 9). The normalized log ratio data and calculated *P*-values from each microarray study were directly uploaded into the database. ChIP-chip DNA binding data for 254 regulators were also compiled from published sources (6, 7), and lists of bound target genes, which were identified based on the published binding criteria (e.g. *P* < 0.005 or log ratio threshold), were uploaded. The Harbison *et al.* ChIP-chip data (6) was first processed in the Ceres database (10) prior to uploading into RegulatorDB.

The RegulatorDB database and website were adapted from a software framework that we previously used for promoter databases for a variety of species, including yeast (10–12). For the Gene Set Overlap tool, the statistical significance is calculated using the cumulative hypergeometric distribution function implemented in the GNU scientific library (13). For the Gene Set Viewer tool, statistical significance is calculated using the Wilcoxon rank sum test, based on code from: www.fon.hum.uva.nl/rob/SignedRank/WlcxTest.pl. The clustering tool was implemented in C ++ using a hierarchical clustering algorithm, with Manhattan distance and complete linkage. Network diagrams for the Regulator Network and Regulator Targets tools are generated using the Cytoscape Web software (14).

## Results and discussion

**Figure 1.**
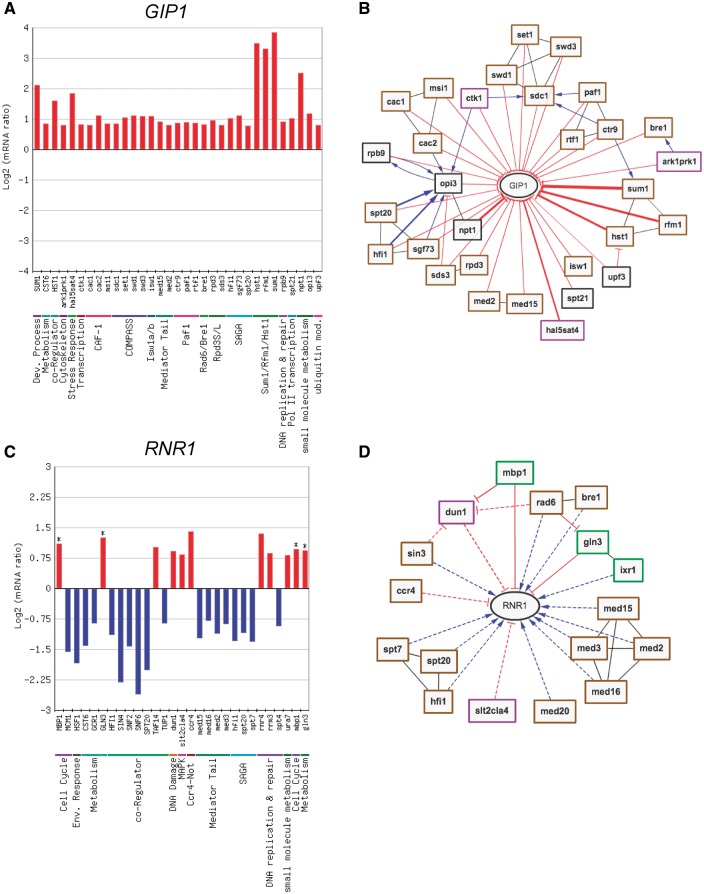
(**A, B**) Analysis and visualization of regulators that control expression of the *GIP1* gene, a sporulation-specific regulator of the Glc7 phosphatase. (A) Output of the Target Viewer tool, displaying changes in *GIP1* expression (log_2_ mRNA ratio) in each regulator mutant that significantly affects *GIP1* expression (fold change >1.7 [up or down] and a *P* < 0.05). Each regulator is grouped by protein complex membership or functional category. (B) Visualization of the network of regulators that regulate *GIP1* transcription (fold change > 1.7 [up or down]; *P* < 0.05). For simplicity, only regulator mutant profiles from yeast grown in SC media conditions are depicted. Blue arrows represent positive regulation; red lines with a cross bar represent negative regulation. Black undirected edges between regulators indicate a shared functional category or complex. (**C, D**) Analysis of regulators that control the expression of the key ribonucleotide reductase gene *RNR1*. (C) Display of changes in *RNR1* expression in each regulator mutant in which *RNR1* is differentially expressed. Asterisks indicate regulators that directly bind to the promoter or coding region of the *RNR1* gene based on published ChIP-chip data. (D) Output of the Regulator Network tool visualizing regulators from the chromatin (brown outline), kinases/phosphatases (purple outline) and transcription factor (SC media) data sets (green outline) that significantly regulate *RNR1* transcription (fold change > 1.5 [up or down]; *P* < 0.05). Same as in part **B**, except solid lines indicate the regulator binds to target gene; dashed lines indicate the target gene is not bound or binding data is not available.

RegulatorDB has six primary tools that can be used to analyze the transcriptional targets of yeast regulators. The Target Viewer tool can be used for rapid visualization of the expression changes of a single target gene in each of the regulator mutants. The Target Viewer tool can be used for rapid visualization of the expression changes of a single target gene in each of the regulator mutants. We used this tool to analyze which regulators significantly affected the expression of the *GIP1/YBR045C gene*, which encodes a sporulation-specific regulator of the Glc7 phosphatase (15). The resulting graphical output depicts the change in mRNA levels of the *GIP1* gene in those regulator mutants that significantly affect *GIP1* expression (Figure 1A). By default, significant targets must have a *P* < 0.05 and fold change >1.7 (up or down) in the regulator mutant, as previously described (1–3); however, many of the RegulatorDB tools allow users to set custom thresholds to define significant targets. The regulators are grouped based on protein complex membership (e.g. COMPASS complex) or functional category (e.g. small molecule metabolism). The Target Viewer tool can also display the changes in mRNA levels of the target gene (e.g. *GIP1*) for all regulator mutants, not just those in which mRNA levels are significantly affected (data not shown). Moreover, the *P*-value and log_2_ ratio of the change in mRNA levels of the target gene for each regulator mutant is included in the resulting output as a downloadable table.

The same expression data for *GIP1* can be represented as a network diagram using the Regulator Network tool (Figure 1B). A number of the negative regulatory relationships depicted in Figure 1B have been previously reported in the literature, including the repression of *GIP1* by the Sum1/Rfm1/Hst1 middle sporulation repressor complex (16). By default, significant targets of regulators must have a *P* < 0.05 and fold change >1.7 (up or down) in the regulator mutant (see above), but the Regulator Network tool allows custom fold-change and *P*-value thresholds to be set for defining regulator target genes. The regulator network output distinguishes between negative/repressive edges (indicated with red lines), in which the target gene is up-regulated in the regulator mutant and positive edges (indicated with blue arrows), in which the target gene is down-regulated in the regulator mutant (Figure 1B). Moreover, the Regulator Network tool provides the option to scale the size of the line/edge based on the magnitude of the gene expression change in the target gene. For example, *GIP1* is most strongly repressed by the Sum1/Rfm1/Hst1 repressor complex (Figure 1A), so these regulatory edges are thicker than for other regulators in the network (Figure 1B). Importantly, regulatory relationships between regulators are also depicted. In this example, the Ctr9 regulator, a subunit of the Paf1 transcription elongation complex, may repress the expression of *GIP1* indirectly, potentially by regulating the expression of Sum1 and/or Sdc1. Other complicated regulatory relationships involving Opi3, Ctk1, etc. are also apparent in the *GIP1* regulator network

The Target Viewer and Regulator Network tools also integrate ChIP-chip DNA binding data in the gene expression network in order to indicate which regulatory relationships involve direct DNA-binding of the regulator to the promoter or coding region of the target gene. To illustrate this functionality, we analyzed the regulation of the *RNR1/YER070W* gene, which encodes the large subunit of the ribonucleotide reductase enzyme that makes deoxynucleotides (dNTPs) for cellular DNA synthesis. Target Viewer analysis identified the regulator mutants in which *RNR1* is differentially expressed (Figure 1C). Only a third of these identified *RNR1* regulators (8 out of 24) were also listed at the Saccharomyces Genome Database (Supplementary Figure S1A). Regulators that bind the *RNR1* promoter or coding sequence are indicated with an asterisk (Figure 1C). These include the transcription factors Mbp1, which is known to regulate *RNR1* expression during G1/S phase (17), and the nitrogen regulator Gln3. Many other transcription factors bind to the *RNR1* promoter or coding sequence (Supplementary Figure S1B), yet most had little to no effect on *RNR1* expression. The Regulator Network tool has an option to represent regulatory interactions/edges using solid lines to indicate bound targets (i.e. ChIP-chip data indicate that promoter or coding region of the target gene is bound by the regulator), while dashed lines indicate targets that are not bound or in which DNA binding data are not available. We used this option to visualize the *RNR1* regulator network; for simplicity, we only visualized regulators in the chromatin (brown outline), kinases/phosphatases (purple outline) and transcription factor (yeast grown in SC media) data sets (green outline) and slightly relaxed the fold change threshold for significant targets (see legend). Inspection of the resulting network indicates, for example, that the regulation by the Mbp1 and Gln3 transcription factors is likely direct because they directly bind the *RNR1* target gene (Figure 1D). Moreover, it is apparent that the Rad6 E2 ubiquitin conjugating enzyme may affect the expression of *RNR1* indirectly by regulating the expression of Gln3 and Dun1 (Figure 1D).

**Figure 2.**
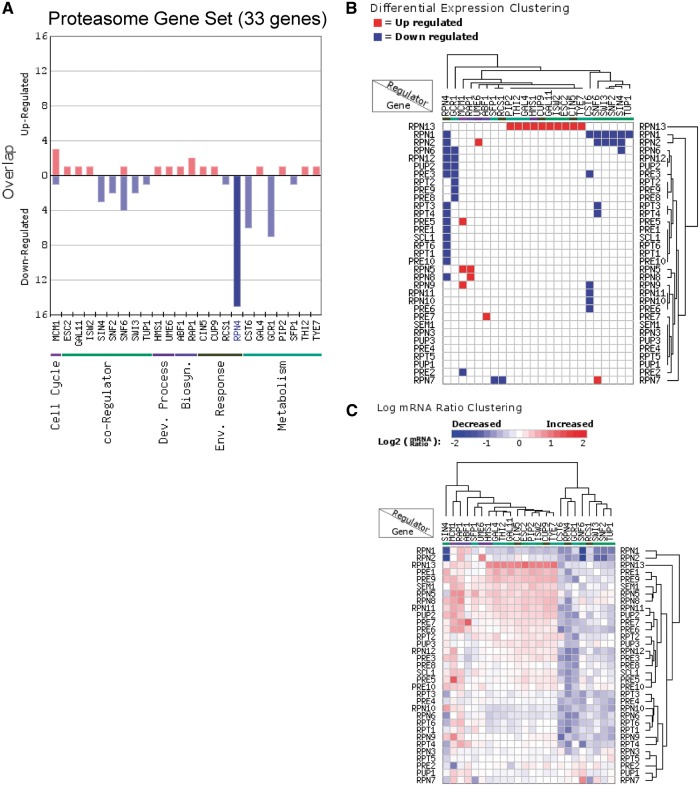
Analysis of the regulation of proteasome genes (33 genes) using the Gene Set Overlap and Regulator Cluster tools. (**A**) Overlap of up- or down-regulated target genes for each regulator in the transcription factor (yeast grown in YPD) category with the proteasome gene set. Only regulators with targets in this gene set are depicted. (**B**) Differential expression clustering, in which the gene expression changes are represented as up-regulated, down-regulated, or unchanged in each regulator mutant for genes in the proteasome gene set. (**C**) Log ratio clustering using the Regulator Cluster tool, in which the clustering is based on the log_2_ mRNA ratio of each gene in each regulator mutant.

In addition to analyzing the regulation of individual genes, RegulatorDB can be used to identify regulators that coordinately control the expression of genes within co-expressed or functional gene sets. We used the Gene Set Overlap tool to analyze the overlap of the proteasome gene set (33 genes) with the sets of target genes for each regulator in the transcription factor/co-activator category (Figure 2A). Targets of the Rpn4 transcription factor (genes down-regulated in the *rpn4Δ* mutant) were found to significantly overlap with the set of proteasome genes (*P* < 10^−24^), indicating that the Rpn4 is required to activate the expression of a number of proteasome genes, in agreement with previous studies (18, 19). The effects of Rpn4 and other regulators on the expression of individual target genes can be visualized using the Regulator Cluster tool, which can be directly accessed from the results page of the Gene Set Overlap tool output. The clustering output for regulators in the transcription factor/co-activator category that significantly affect the expression of gene(s) in the proteasome gene set is shown in Figure 2B. This particular visualization displays and clusters the genes based on whether they are differentially expressed (either up- or down-regulated) in each regulator mutant. Inspection of the clustering data indicate that the genes encoding the Rpn13, Rpn1 and Rpn2 proteasome subunits are differentially expressed in a relatively large number of regulator mutants relative to other proteasome subunits (Figure 2B). Interestingly, Rpn13 and Rpn1 play particularly important roles in recognition of ubiquitylated substrates by the 19 S regulatory particle of the proteasome, which could explain why the expression of these genes is more highly regulated (20, 21). The Regulator Cluster tool can also cluster target genes based on the actual log ratio of the change in mRNA levels in each regulator mutant (Figure 2C).

**Figure 3.**
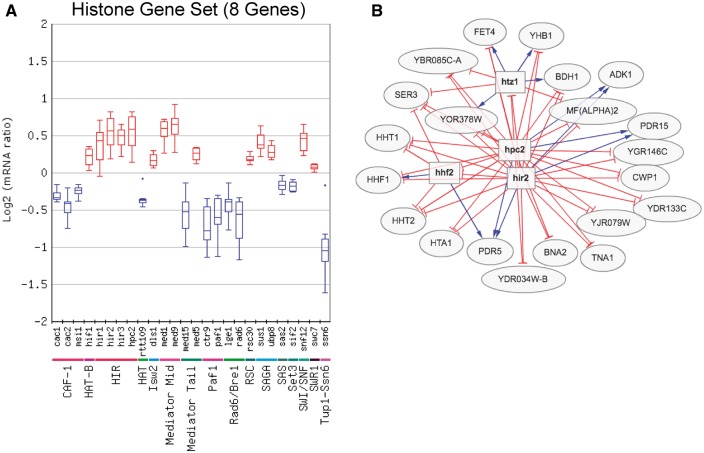
(**A**) Box plot output of the Gene Set Viewer tool, displaying changes in the expression of the core histone genes (*HTA1*, *HTA2*, *HTB1*, *HTB2*, *HHF1*, *HHF2*, *HHT1* and *HHT2*). A box plot depicting the log_2_ mRNA ratios of the histone genes for each regulator mutant in the chromatin regulator category is depicted. Only regulator mutants that significantly affect the expression of the histone gene set (calculated using Wilcoxon rank sum test, see methods) are displayed. Regulators are grouped by protein complex or functional category. (**B**) Target genes regulated by both the Hir2 and Hpc2 subunits of HIR histone chaperone and repressor complex, using a threshold of *P* < 0.05 and fold change >1.3 (up or down). Target genes were identified using the Regulator Targets tool, and the depicted network display is adapted from the output of this tool.

The Gene Set Viewer tool, which displays the log mRNA expression ratios of a set of genes as a box plot or average percentile, is an alternative method for analyzing regulator/gene set associations. Figure 3 shows an example of the box plot display of the expression changes of the eight core histone genes for the chromatin regulator category, in this case depicting only regulators in which the expression of the histone genes was significantly altered in the regulator mutant. Visualization of the expression changes of the histone genes in all chromatin regulator mutant profiles is shown in Figure 3. This analysis identified many known regulators of histone gene expression, such as the HIR complex [Hir1, Hir2, Hir3 and Hpc2 (22)], as well as number of potential novel regulators of histone expression. These include a number of factors involved in chromatin assembly, such as subunits of the chromatin assembly factor-I (CAF-I) complex and Rtt109 histone acetyltransferase (Figure 3). It is possible that histone gene transcription is reduced in these mutants due to their defects in chromatin assembly, in order to avoid the accumulation of excess free histones, which can induce genome instability and is generally toxic to cells (23, 24). Importantly, the Gene Set Viewer tool uses a sensitive method (the non-parametric Wilcoxon Rank Sum test) to detect significant associations with regulators, and thus can detect regulator-target gene associations that are relatively subtle or small in magnitude but are consistent across a set of co-regulated genes. For example, many of the changes in histone gene expression in these regulator mutants did not meet the typical threshold for significance [i.e. *P* < 0.05 and fold change >1.7 (up or down)], yet were detected by the Gene Set Viewer tool (Figure 3A).

The Regulator Targets tool displays all of the target genes whose expression is significantly affected by a user-selected regulator or set of regulators. Again, the user can define the *P*-value and fold change threshold used for target gene identification. The Regulator Targets tool was used to visualize target genes repressed by the Hir2 and Hpc2 subunits of the HIR complex in yeast grown in SC media using AND logic. Since many of the target genes of the HIR complex, such as the histone genes, showed relatively subtle changes in gene expression, a threshold of *P* < 0.05 and fold change >1.3 (up or down) was chosen for this analysis. The resulting output (Figure 3B) revealed many known targets, including most of the canonical histone genes (i.e. *HTA1*, *HHF1*, *HHF2*, *HHT1*, *HHT2*). Of the histone genes not detected as targets, *HTB1* just barely missed the fold change threshold for Hpc2 (data not shown), and the *HTA2*-*HTB2* gene pair was previously shown not to be regulated by the HIR complex (25). A number of novel targets of Hir2 and Hpc2 were also identified, including the histone variant *HTZ1* (Figure 3B).

In summary, we anticipate that the RegulatorDB database will have significant utility for elucidating the regulation of individual genes, gene sets and genetic pathways in the widely used model eukaryote *S. cerevisiae*. Importantly, by integrating DNA binding data and mutant expression profiles in a user-friendly manner, the RegulatorDB analysis tools could greatly facilitate the study of transcriptional regulatory networks in this important model organism.

## Supplementary data

Supplementary data are available at *Database* Online.

## Supplementary Material

Supplementary Data
