# Supplementary material for: RegulatorDB: a resource for the analysis of yeast transcriptional regulation
Source: Database (Oxford). 2017 Aug 3;2017:bax058. doi: 10.1093/database/bax058 (PMC5737240; doi:10.1093/database/bax058)

SUPPLEMENTAL MATERIALS

Supplemental Figure Legends

Supplemental Figure 1. (**A**) Network of regulators controlling *RNR1* expression from the Saccharomyces Genome Database (1). *SIN4* is a synonym for *MED16*. (**B**) Changes in *RNR1* expression (log2 mRNA ratio) in each regulator mutant that binds to the *RNR1* promoter or coding region (indicated by asterisk), based on published ChIP-chip data. Each regulator is grouped by protein complex membership or functional category.

Supplemental References

1. Costanzo, M.C., Engel, S.R., Wong, E.D., Lloyd, P., Karra, K., Chan, E.T., Weng, S., Paskov, K.M., Roe, G.R. and Binkley, G. (2014) Saccharomyces genome database provides new regulation data. *Nucleic acids research*, **42**, D717-D725.

Supplemental Figure 1

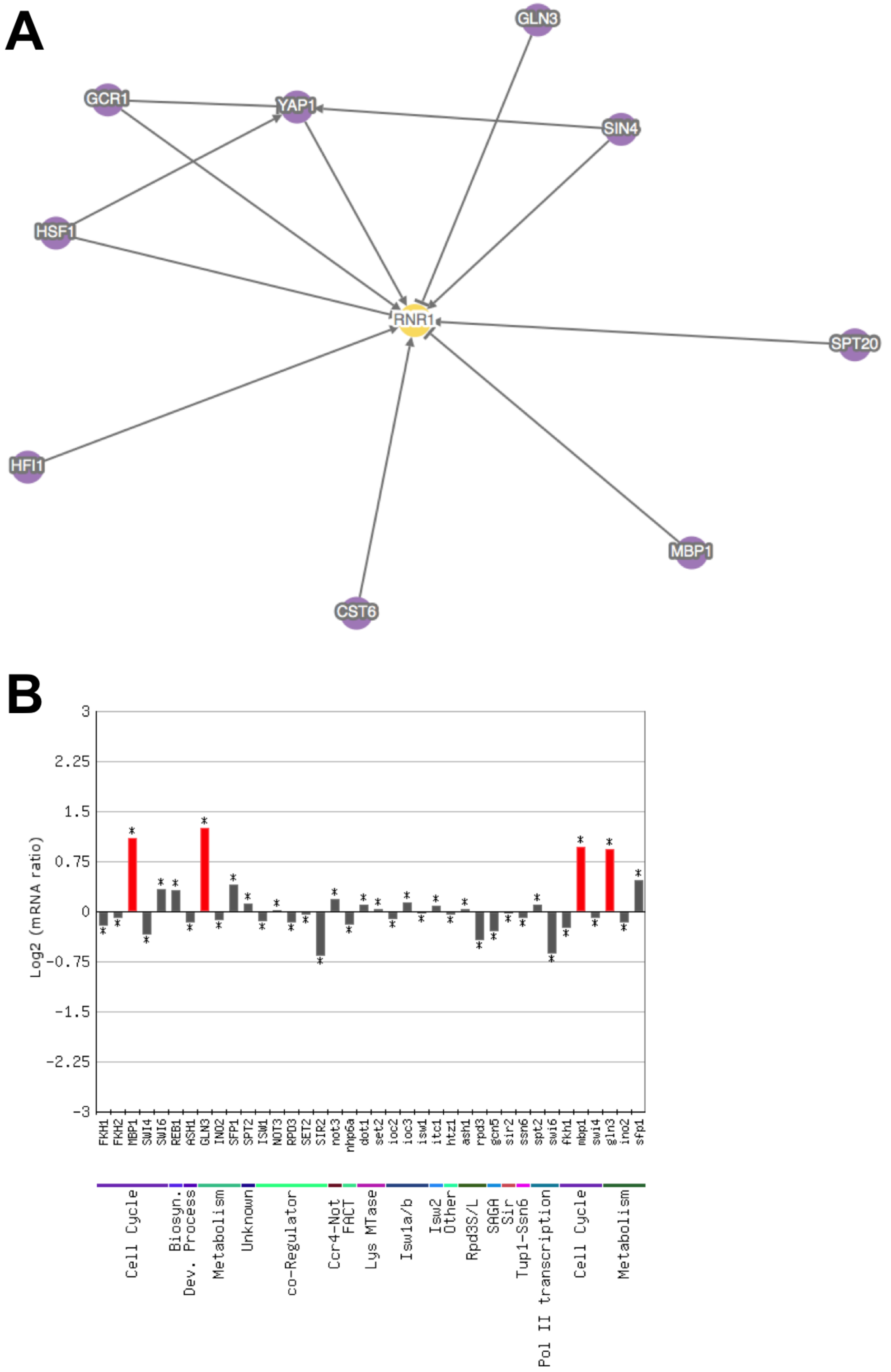

Supplement: Supplementary Data [file bax058_supp.pdf]
